# Dznep, a histone modification inhibitor, inhibits HIF1α binding to TIMP2 gene and suppresses TIMP2 expression under hypoxia

**DOI:** 10.14814/phy2.15810

**Published:** 2023-09-14

**Authors:** Tomotaka Yamazaki, Imari Mimura, Yu Kurata, Tetsuhiro Tanaka, Masaomi Nangaku

**Affiliations:** ^1^ Division of Nephrology and Endocrinology The University of Tokyo Graduate School of Medicine Tokyo Japan; ^2^ Department of Nephrology, Rheumatology and Endocrinology Tohoku University Graduate School of Medicine Sendai Japan

**Keywords:** Dznep, hypoxia‐inducible factor, open chromatin

## Abstract

Epidemiological studies have shown that patients who recovered from acute kidney injury (AKI) may subsequently develop chronic kidney disease (CKD). AKI is primarily caused by renal hypoxia, and it causes epigenetic alterations, known as hypoxic memory. 3‐Deazaneplanocin A (Dznep), an inhibitor of histone modification, suppresses renal fibrosis and the expression of tissue inhibitor of metalloproteinases‐2 (TIMP2), a profibrotic factor, in mouse ischemia–reperfusion models. The current study investigated the epigenetic regulation of TIMP2 in human kidney 2 (HK‐2) cells. The expression of TIMP2 was upregulated in HK‐2 cells under hypoxic conditions and was suppressed by Dznep. ChIP‐qPCR showed that Dznep reduced the amount of H3K4me3 at the promoter region of the TIMP2 gene under hypoxic condition. Formaldehyde‐assisted isolation of regulatory elements‐qPCR of the TIMP2 gene showed that Dznep reduced open chromatin area. In addition, based on ChIP‐qPCR of hypoxia‐inducible factor 1 alpha (HIF1α), Dznep inhibited the binding of HIF1α to the TIMP2 gene under hypoxic conditions. The reporter assays for the binding region of HIF1α showed enhanced transcriptional activity by hypoxia. Dznep suppresses the expression of TIMP2 under hypoxic conditions by inhibiting the binding of HIF1α to the TIMP2 gene.

## Abstract 2

We previously reported that the TIMP2, a profibrotic factor, was elevated in ischemia–reperfusion mice and was suppressed by Dznep. However, the epigenetic mechanism by which TIMP2 was suppressed by Dznep has not been elucidated. In this study, we showed that hypoxia increased H3K4me3 and open chromatin in the TIMP2 gene and increased HIF1α binding, and Dznep suppressed TIMP2 expression by reversely inhibiting these changes.

## 1 INTRODUCTION

Acute kidney injury (AKI) was previously believed to be a transient condition with complete recovery of renal function. However, recent epidemiological studies have reported that some patients who completely recovered from AKI develop chronic kidney disease (CKD) and, eventually, end‐stage kidney disease. This phenomenon is referred to as AKI‐to‐CKD transition (Coca et al., 2012) and is primarily caused by renal hypoxia. AKI is stored in cells as hypoxic memory, which is the epigenetic alteration caused by transient hypoxia (Mimura, 2016; Nangaku et al., 2017; Tanemoto & Mimura, 2022). In diabetic kidney disease, transient hyperglycemia is stored as epigenetic cellular memory, referred to as metabolic memory, which causes the progression of diabetic kidney disease (Yamazaki et al., 2021). Irreversible kidney damage progresses to CKD regardless of cause, and the vicious cycle of tubular interstitial hypoxia is the final common pathway associated with progression (Mimura et al., 2013; Mimura & Nangaku, 2010; Nangaku, 2006). Hence, epigenetics and hypoxia are important factors in the development of AKI‐to‐CKD and the subsequent progression of CKD.

Dznep is an inhibitor of the expression of zeste homolog 2 (Ezh2), which methylates H3K27me3, a gene repressor histone marker (Miranda et al., 2009). Further, it reduces renal fibrosis in mice with unilateral ureteral obstruction (UUO) by maintaining the expression of Smad7 and phosphatase and tensin homolog (PTEN) (Zhou et al., 2016). It has renoprotective effects in not only renal fibrosis models but also AKI models, such as ischemia/reperfusion (I/R) injury and cisplatin nephropathy, and hyperuricemia and acute rejection models (Li et al., 2016; Ni et al., 2019; Shi et al., 2019; Zhou et al., 2018). Our previous study found that Dznep reduced renal fibrosis in a mouse AKI‐to‐CKD model with long‐term I/R injury. Moreover, there was elevated expression of tissue inhibitor of metalloproteinase 2 (TIMP2), a pro‐fibrotic factor, in I/R injury. However, this mechanism was suppressed by Dznep (Mimura et al., 2018). The drug inhibits not only Ezh2 but also S‐adenosylhomocysteine hydrolase. Inhibiting S‐adenosylhomocysteine hydrolase reduces the methyl groups by suppressing the methionine circuit. In breast cancer cell lines, Dznep inhibits the methylation of histones other than H3K27me3 (Miranda et al., 2009). Although Dznep have different epigenetic effects, previous reports on kidney disease and Dznep did not perform a detailed investigation of the epigenetic changes caused by this drug.

TIMP2 contributes to the development of fibrosis by inhibiting the function of matrix metalloproteinases and by suppressing the degradation of extracellular matrix (Gomez et al., 1997). In the kidney fields, TIMP2, which is an early biomarker of AKI, has attracted attention. For example, the product of TIMP2 and insulin‐like growth factor‐binding protein 7 is an indicator of AKI severity (Kashani et al., 2013). In a previous experiment, sepsis‐induced AKI is associated with less renal damage via the inhibition of the nuclear factor (NF)‐κB pathway in TIMP2 knockout mice (Li et al., 2019). However, there are only few reports about the role of TIMP2 in renal fibrosis and CKD progression. A previous report has shown that UUO‐induced renal fibrosis was suppressed in TIMP2 knockout mice. However, the detailed function of TIMP2 in tubular cells has not been investigated (Wang et al., 2014).

TIMP2 is a factor of renal fibrosis factor. However, its epigenetic regulation is not well understood. Therefore, the current study investigated the molecular mechanism underlying the epigenetic regulation of TIMP2 with Dznep treatment.

## 2 METHODS AND METHODS

### 2.1 Western blot

Histone protein was extracted using the Histone Extraction Kit (Active Motif, California, USA) based on the manufacturer's instructions. The concentration of extracted proteins was assayed using the Pierce™ BCA Protein Assay Kit (Thermo Fisher Scientific, Santa Clara, CA, the USA). Dithiothreitol at a final concentration of 2.5 mM was added to the extracted proteins and warmed in a heat block at 95°C for 5 min. The adjusted proteins were then applied to the sodium dodecyl sulfate (SDS)–polyacrylamide gel for electrophoresis. Next, the proteins were transferred into the polyvinylidene difluoride membrane (GE Healthcare, the UK). The membrane was blocked with 5% skim milk for 30 min at room temperature. After cutting off the unwanted membrane except for the target molecular weight, the membrane was incubated with the primary antibodies (anti‐H3K4me3 antibody and anti‐H3 antibody overnight at 4°C. The membranes were then incubated with secondary antibodies for 30 min at room temperature. The antibodies and concentrations used are listed in Table 2. Pierce ECL Plus Substrate (Thermo Fisher Scientific, Santa Clara, CA, the USA) was applied to detect signals. The band intensity was quantitated using ImageJ (NIH, Bethesda, the USA). The densities of the H3K4me3 bands were corrected for H3 and then quantified by comparing with those of the bands under normoxic condition without Dznep treatment.

### 2.2 RNA isolation, reverse transcription, and qPCR

RNA was isolated using RNAiso Plus (Takara Bio Inc., Shiga, Japan) based on the manufacturer's instructions. Reverse transcription was performed using the PrimeScript RT Master Mix (Takara Bio Inc.). First‐strand cDNA was used to determine the relative mRNA expression with THUNDERBIRD SYBR QPS‐201 (Toyobo Co., Ltd., Osaka, Japan) on the CFX96 System (Bio‐Rad Laboratories, Inc., Hercules, CA, the USA). We have only done up to 39 cycles in RT‐PCR. The expression level of each gene was normalized by β‐actin. All measurements were performed in triplicate, and three independent experiments (*n* = 3) were conducted. Table 1 shows the primer sequences used in qPCR.

**TABLE 1:** List of primers.

Name	Species		
RT‐qPCR			
ACTB	Human	Forward	TCCCCCAACTTGAGATGTATGAAG
		Reverse	AACTGGTCTCAAGTCAGTGTACAGG
TIMP2	Human	Forward	TCCTCTTGATAGGGTTGCCA
		Reverse	CGTTTTGCAATGCAGATGTA
Ezh2	Human	Forward	TGCTTCCTACATCGTAAGTGC
		Reverse	GGACGTTTTGGTGGGGTCTT
Chip‐qPCR			
TIMP2‐1	Human	Forward	CCGAATGTTCCGCTTTTTCG
		Reverse	ATTTGCTGGGGAATCTGAGC
TIMP2‐2	Human	Forward	CCAGAGCTGAGTGGTTCACTG
		Reverse	CCTCGAGTCAGGATAGACCC
HIF1α binding site	Human	Forward	AGTGTGACGAAACTGAGCTGG
		Reverse	CCCGTGGTGCAGGGAACTTA
TIMP2‐4	Human	Forward	CCAGAGCTGAGTGGTTCACTG
		Reverse	CCTCGAGTCAGGATAGACCC
FAIRE‐qPCR			
TIMP2‐3	Human	Forward	ATTGGTGCAACTGAAAACTTCCC
		Reverse	TGGGACTTTCTGTTTCTCCTGC
HIF1α binding site	Human	Forward	AGTGTGACGAAACTGAGCTGG
		Reverse	CCCGTGGTGCAGGGAACTTA
Clonig			
TIMP2‐HIF1α	Human	Forward	ATCCTGTATTCTTGAGCTATTACCCTCCTG
		Reverse	GCAAACAGAGGAACAGGGAATTATTGG
siRNA			
siEzh2‐1	Human	Sense	GACCACAGUGUUACCAGCAUUUGGA
		Antisense	UCCAAAUGCUGGUAACACUGUGGUC
siEzh2‐2	Human	Sense	GAGCAAAGCUUACACUCCUUUCAUA
		Antisense	UAUGAAAGGAGUGUAAGCUUUGCUC

**TABLE 2:** List of antibodies.

Name	Vendor	Cat no.	Dilution
Primary antibody			
H3K4me3	GeneTex	MABI0304	1/500
H3	Cell signaling	9715S	1/1000
Secondary antibody			
Rabbit IgG	BioRad	170–6515	1/10000
Mouse IgG	BioRad	170–6516	1/10000

### 2.3 Cell culture

Human kidney 2 (HK‐2) cells were used in in vitro experiments (CRL‐2190, ACTT, Manassas, VA, the USA). The cells were cultured in Dulbecco's Modified Eagle Medium (Sigma Aldrich) with 10% fetal bovine serum (Sigma Aldrich) and 1% penicillin/streptomycin (Thermo Fisher Scientific). Next, they were cultured in a humidified atmosphere with 5% CO_2_ at 37°C. Hypoxic condition was established via exposure to 1% O_2_ in a hypoxic cultivation incubator (APM‐30D, ASTEC, Fukuoka, Japan). It is noteworthy that exposure of HK2 cells to 1% hypoxic condition for 24 h does not damage cell viability as assessed by the MTS assay (Promega G3582). Dznep was added to the medium at a concentration of 10 μM 24 h prior to hypoxic stimulation in all experiments.

### 2.4 siRNA

HK‐2 cells were passaged in six wells with 1 × 10^5^ cells. After 24 h, siRNAs with lipofectamine RNAiMAX (Thermo Fisher Scientific) were transfected into HK‐2 cells. Stealth RNAi siRNA targeting human Ezh2 and negative control nucleotide (Thermo Fisher Scientific) were used. After 24 of transfection, hypoxic stimulation, Dznep treatment, and assays were initiated.

### 2.5 ChIP and FAIRE

HK‐2 cells stimulated via hypoxia for 24 h and treated with Dznep were collected with trypsin and fixed with 1% formaldehyde for 10 min at room temperature. The fixed cells were lysed in SDS lysis buffer (10‐mM Tris–HCL [pH 8.0], 150‐mM NaCl, 1% SDS, and 1‐mM EDTA), sonicated by Branson Sonifier Cell Disruptor 350 (Branson Ultrasonics Corp., Danbury, CT, the USA), and electrophoresed on agarose gel to validate the DNA fragmentation status.

In the procedure of chromatin immunoprecipitation (ChIP), the primary antibodies were anti‐H3K4me3 antibody (MABI0304, diluted 1:100) (GeneTex, Funakoshi Co., Ltd.) and anti‐HIF1α (hypoxia‐inducible factor 1 alpha) antibody (NB100‐479, diluted 1:50) (Novus Biologicals, Centennial, CO, the USA). Next, they were reacted with Dynabead M‐280 Sheep Anti‐Mouse IgG or Dynabead M‐280 Sheep Anti‐Rabbit IgG (Thermo Fisher Scientific) at 4°C overnight. The reacted beads and the sonicated samples were mixed and rotated at 4°C overnight. The reacted beads were washed and then de‐crosslinked at 65°C overnight. After treatment with Pronase (Roche) and RNase A (QIAGEN, Venlo, the Netherlands), the DNAs were extracted via phenol chloroform extraction and quantified via qPCR as the ratio of input (%input). All measurements were performed in triplicate, and three independent experiments (*n* = 3) were conducted.

In the procedure of FAIRE (formaldehyde‐assisted isolation of regulatory elements), the input samples were de‐crosslinked at 65°C overnight and treated with Pronase. In all samples including input samples, DNAs were extracted via phenol chloroform extraction after RNase A treatment. The purified DNAs were quantified via qPCR as %input. Then, the value of %input was standardized as the ratio to the negative control area. Table 1 shows the sequences of the primers used in qPCR. All measurements were performed in triplicate, and three independent experiments were conducted.

### 2.6 Reporter assay for the HIF1α‐binding site of the TIMP2 gene

The HIF1α‐binding site of the TIMP2 gene was extracted from our previous HIF‐1α ChIP‐seq data (Kushida et al., 2016). The gene area of this region was amplified via PCR with the primers shown in Table 1. The PCR product was electrophoresed in agarose gel, and the HIF1α‐binding DNA was extracted with the QIAquick Gel Extraction Kit (QIAGEN). The HIF1α‐binding DNA was subcloned to the Zero Blunt TOPO vector using the Zero Blun TOP PCR Cloning Kit (Thermo Fisher Scientific). Competent high JM109 (TOYOBO) transformed by the TOPO vector was cultivated on the Kanamycin plate. Miniprep was performed on the cultured colonies using the PureYield™ Plasmid Miniprep System (Promega, Madison, WI, the USA), and the correctly cloned plasmid was confirmed based on the DNA sequences. Then, the TOPO vector and pGL3‐promotor vector (Promega) were cut using restriction enzymes (KpnI and XhoI; Takara Bio Inc.) and ligated with Mighty Mix (Takara Bio Inc.). The ligated vector was transformed into JM109. Next, JM109 transformed by the ligated vector was cultivated on the ampicillin plate. After performing colony PCR, midiprep was performed on the cultured colonies using the PureYield™ Plasmid Midiprep System (Promega). Then, we obtained the TIMP2‐HIF1α‐binding DNA inserted into the pGL3‐promotor vector (TH vector).

HK‐2 cells were passaged in 12 wells with 5 × 10^4^ cells. After 24 h, the TH vector or the pGL3‐promotor vector with Fugene HD (Promega) were transfected into HK‐2 cells. After 24 h of transfection, hypoxic stimulation was initiated. After 24 h, the assay was started using the Dual‐Luciferase Reporter Assay System (Promega) based on the manufacturer's instructions. Fluorescence values were used as the ratio of Firefly and Renilla measurements. All measurements were performed in triplicate, and three independent experiments (*n* = 3) were conducted.

### 2.7 Statistical analysis

All the data are reported as mean ± standard error of mean and as individual values in the dot plots. The unpaired two‐tailed *t*‐test was conducted to analyze data between two groups. *p*‐values of <0.05 were considered statistically significant. All analyses were performed with GraphPad Prism version 9.3.1 (GraphPad Software Inc.).

## 3 RESULTS

### 3.1 The expression of TIMP2 is upregulated by hypoxic stimulation and suppressed by Dznep treatment in HK‐2 cells

**FIGURE 1:**
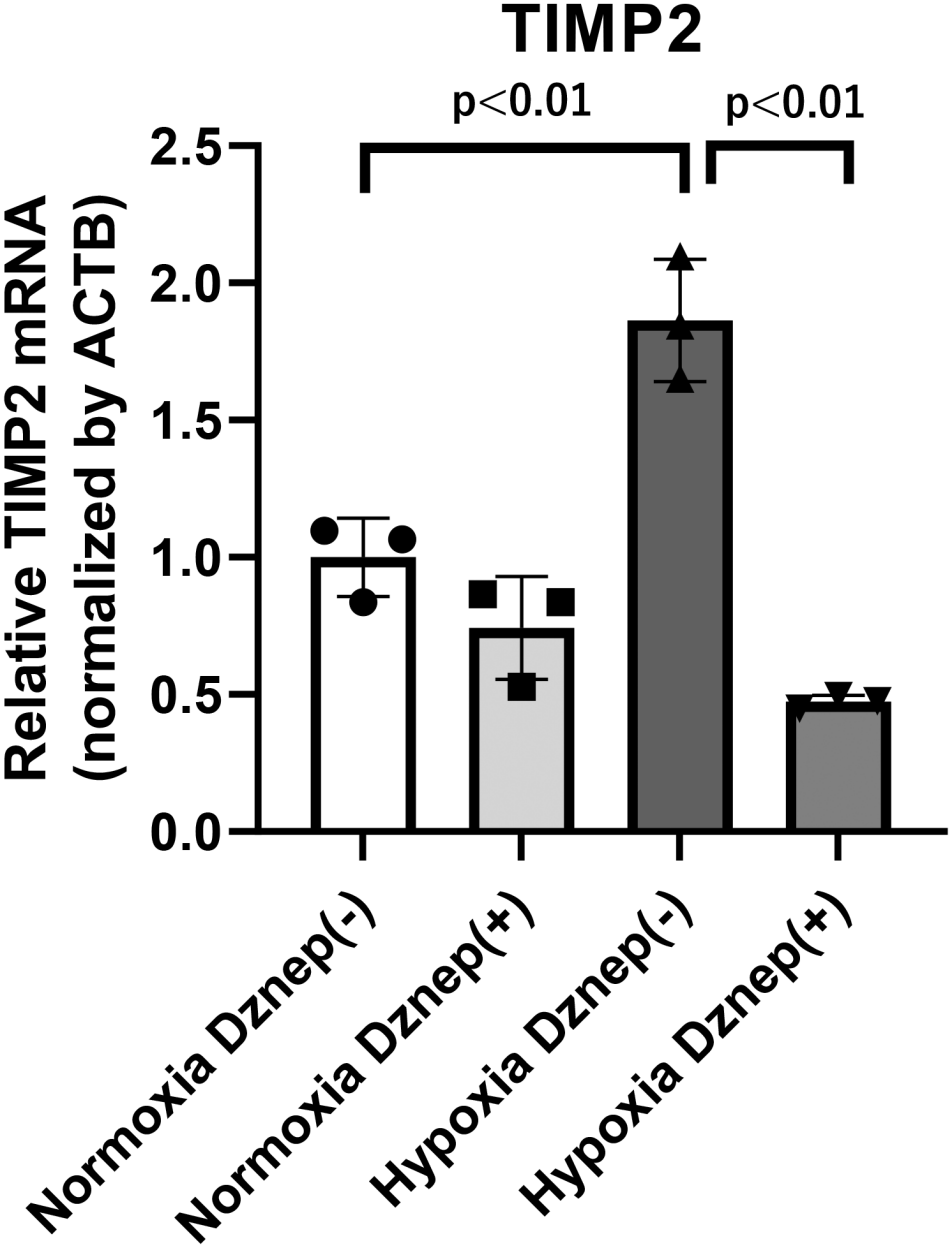
The mRNA expression of TIMP2 in HK‐2 cells under normoxic condition and hypoxic condition for 24 h with or without Dznep treatment. First bar and plots indicate normoxia without Dznep (1.00 ± 0.142). Second bar and plots indicate normoxia with Dznep (0.743 ± 0.187). Third bar and plots indicate hypoxia without Dznep (1.864 ± 0.223). Fourth bar and plots indicate hypoxia with Dznep (0.474 ± 0.023). ACTB, beta actin; Dz, Dznep; HK‐2, human kidney 2; TIMP2, tissue inhibitor of metalloproteinase 2. Three independent experiments (*n* = 3) were conducted.

HK‐2 cells were cultured under 1% hypoxic condition for 24 h. We measured mRNA level of *VEGF* to confirm that hypoxic stimulation was effective because *VEGF* is one of the well‐known genes which are upregulated under hypoxic condition compared with normoxia. The mRNA level of *VEGF* with Dznep is upregulated (2.10 ± 0.31 fold) and the mRNA level of *VEGF* without Dznep is also significantly upregulated (2.58 ± 0.59 fold) (Figure S1). The mRNA level of TIMP2 was significantly upregulated under hypoxia without Dznep (1.86 ± 0.22 fold) and significantly inhibited by Dznep treatment (0.25 ± 0.012 fold) (Figure 1) compared to that without Dznep under hypoxic condition.

### 3.2 Dznep reduces H3K4me3 in HK‐2 cells

**FIGURE 2:**
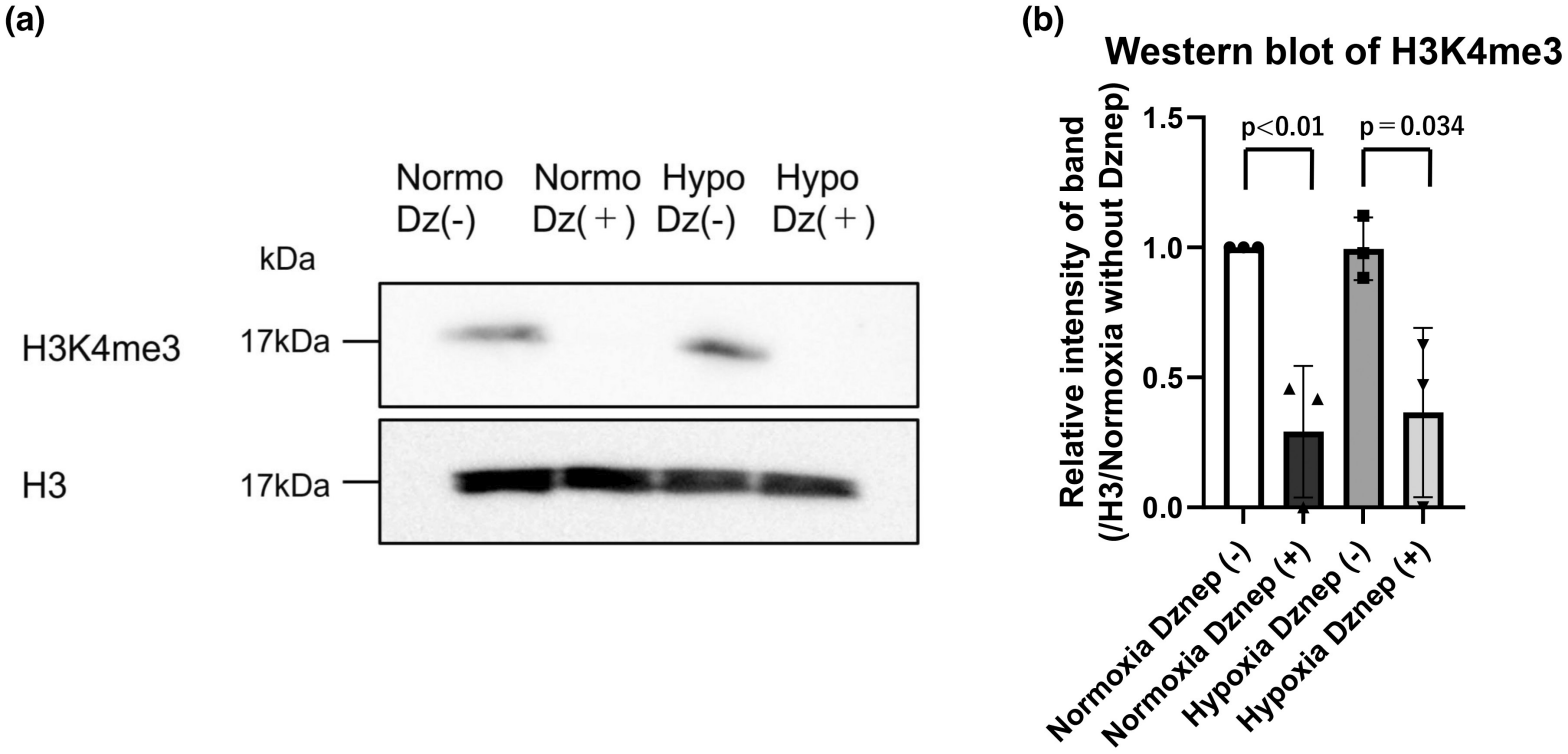
Western blot of H3K4me3 extracted from HK‐2 cells under normoxic condition and hypoxic condition for 24 h with or without Dznep treatment (a). Quantitative evaluations of the density of these H3K4me3 bands (b). The band intensity was quantitated using ImageJ (NIH, Bethesda, the USA). The densities of the H3K4me3 bands were corrected for H3 and then quantified by comparing with those of the bands under normoxic condition without Dznep treatment. The cropped blots were used in the figure and different membranes were separated by lines. First bar and plots indicate normoxia without Dznep. Second bar and plots indicate normoxia with Dznep (0.456 ± 0.253). Third bar and plots indicate hypoxia without Dznep (1.122 ± 0.120). Fourth bar and plots indicate hypoxia with Dznep (0.624 ± 0.325). All the blots we have done are presented in Figure S3. Dz, Dznep; HK‐2, human kidney 2; Hypo, hypoxia; Normo, normoxia; WB, western blot. WB was performed three times (*n* = 3) as an independent experiment.

We examined whether Ezh2 regulates TIMP2 in HK‐2 cells. We used siRNA of Ezh2 to examine the downstream genes of Ezh2. The knockdown efficiency is 94.9 ± 5.1% (Ezh2 si#1) and 71.1 ± 2.0% (Ezh2 si#2) under normoxia and 95.1 ± 4.8% (Ezh2 si#1) and 69.6 ± 2.0% (Ezh2 si#2) under hypoxia (Figure S2a). The results of our experiments showed that suppression of Ezh2 by siRNA did not significantly alter the expression level of TIMP2 (Figure S2b) under both normoxic and hypoxic conditions. These results suggested that histone modifications other than H3K27me3 may be involved in TIMP2 expression in HK‐2 cells. To investigate the effects of Dznep aside from H3K27me3 and Ezh2 suppression, we examined the other types of histone methylation. According to ENCODE's ChIP sequencing data, H3K4me3 is accumulated in the promoter region of TIMP2. H3K4me3 is a transcriptional marker and is located in the promoter region of active genes. Western blot using H3K4me3 antibody was performed to examine the methylation status of histones in HK‐2 cells (Figure 2a). Full membranes of WB are shown in Figure S3a. The experiments were independently performed three times and other membranes are shown in Figure S3b. From the results in Figure 2a,b, we confirmed that Dznep suppressed the methylation of H3K4me3 both under normoxia and hypoxic conditions.

### 3.3 Dznep decreases H3K4me3 methylation and open chromatin area in the TIMP2 gene region under hypoxic condition

**FIGURE 3:**
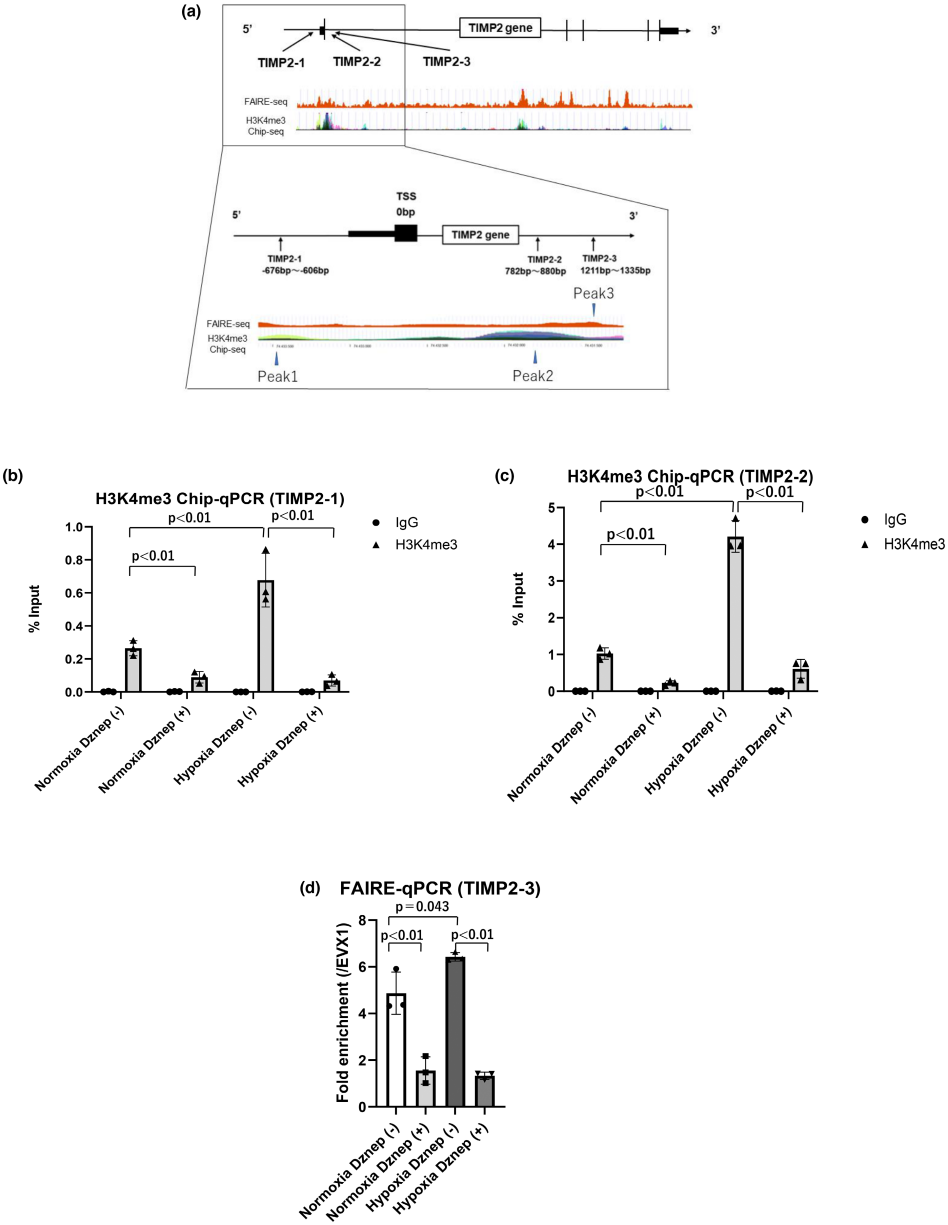
ChIP‐qPCR of H3K4me3 and FAIRE‐qPCR of the TIMP2 gene in HK‐2 cells under normoxic condition and hypoxic condition for 24 h with or without Dznep treatment. The H3K4me3 target regions and FAIRE target region of the TIMP2 gene were based on the ENCODE ChIP‐seq data and FAIRE‐seq data (a). In addition, the FAIRE‐seq data (which were submitted to ENCODE on September 23, 2009) revealed FAIRE‐seq peaks in HUVEC cells under normoxic condition. The results of ChIP‐qPCR of H3K4me3 in peak1(TIMP2‐1) were evaluated using %input. First bar and plots indicate normoxia using normal IgG without Dznep (0.00143 ± 0.00248). Second bar and plots indicate normoxia using anti‐H3K4me3 antibody without Dznep (0.264 ± 0.045). Third bar and plots indicate normoxia using normal IgG with Dznep (0.00178 ± 0.00166). Fourth bar and plots indicate normoxia using anti‐H3K4me3 antibody with Dznep (0.089 ± 0.034). Fifth bar and plots indicate hypoxia using normal IgG without Dznep (0.000181 ± 0.000314). Sixth bar and plots indicate hypoxia using anti‐H3K4me3 antibody without Dznep (0.677 ± 0.162). Seventh bar and plots indicate hypoxia using normal IgG with Dznep (0.000953 ± 0.000826). Eighth bar and plots indicate hypoxia using anti‐H3K4me3 antibody with Dznep (0.069 ± 0.034) (b). The results of ChIP‐qPCR of H3K4me3 in peak2 (TIMP2‐2) were evaluated using %input. First bar and plots indicate normoxia using normal IgG without Dznep (0.00124 ± 0.00119). Second bar and plots indicate normoxia using anti‐H3K4me3 antibody without Dznep (1.027 ± 0.154). Third bar and plots indicate normoxia using normal IgG with Dznep (0.00124 ± 0.00118). Fourth bar and plots indicate normoxia using anti‐H3K4me3 antibody with Dznep (0.226 ± 0.059). Fifth bar and plots indicate hypoxia using normal IgG without Dznep (0.000310 ± 0.000537). Sixth bar and plots indicate hypoxia using anti‐H3K4me3 antibody without Dznep (4.212 ± 0.429). Seventh bar and plots indicate hypoxia using normal IgG with Dznep (0.00142 ± 0.00246). Eighth bar and plots indicate hypoxia using anti‐H3K4me3 antibody with Dznep (0.612 ± 0.255) (c). The finding of FAIRE‐qPCR was assessed via fold enrichment over the EVX1 region, which is the negative control region. First bar and plots indicate normoxia without Dznep (4.870 ± 0.906). Second bar and plots indicate normoxia with Dznep (1.556 ± 0.585). Third bar and plots indicate hypoxia without Dznep (6.429 ± 0.182). Fourth bar and plots indicate hypoxia with Dznep (1.332 ± 0.155) (d). EVX1, even‐skipped homeobox 1; FAIRE, formaldehyde‐assisted isolation of regulatory elements; HK‐2, human kidney 2; TIMP2, tissue inhibitor of metalloproteinase 2; TSS, transcriptional start site. *p < 0.05, **p < 0.01. Three independent experiments (*n* = 3) were conducted.

The effects of hypoxia and Dznep treatment on the methylation of H3K4me3 in the TIMP2 gene region were examined via ChIP‐quantitative real‐time polymerase chain reaction (qPCR). There are two peaks (peak 1, peak 2) of H3K4me3 based on ChIP‐seq data of ENCODE data (Figure 3a). We made the two ChIP‐qPCR primers for the TIMP2 gene ENCODE (peak 1; TIMP2‐1, peak 2; TIMP2‐2). ChIP‐qPCRs using H3K4me3 antibodies were performed on these two regions. In both regions, the amount of H3K4me3 were elevated in hypoxic condition (2.87 ± 0.87 fold; (TIMP2‐1) Figure 3b), (3.86 ± 0.003 fold; (TIMP2‐2) Figure 3c) and this elevation was significantly suppressed by the treatment of Dznep (0.11 ± 0.03 fold; (TIMP2‐1) Figure 3b), (0.18 ± 0.001 fold; (TIMP2‐2) Figure 3c). These results suggested that Dznep inhibited the methylations of H3K4me3 in the promoter region of TIMP2. Further, the open chromatin area was investigated via FAIRE‐qPCR. We found a peak (peak 3 in Figure 3a) near the H3K4me3 region (peak 2) from the FAIRE‐seq data of ENCODE which were submitted to ENCODE on September 23, 2009 (Figure 3a). Since FAIRE can detect open chromatin, where histone does not exist as shown in Figure 3a. According to the results of FAIRE‐seq, we made a primer of TIMP2‐3. ENCODE included FAIRE‐seq peaks in HUVEC cells under normoxic condition. We performed FAIRE‐qPCR in this region. Similar to the results for H3K4me3, the fold enrichment compared to the EVX1 (negative control gene) significantly increased (1.32 ± 0.037 fold) under hypoxic condition and was suppressed (0.21 ± 0.024 fold) by Dznep (Figure 3d). This result showed that Dznep inhibited open chromatin regions in addition to H3K4me3 in the promoter region of TIMP2.

### 3.4 Dznep inhibits the binding of HIF1α to the TIMP2 gene region by reducing open chromatin area under hypoxic conditions

**FIGURE 4:**
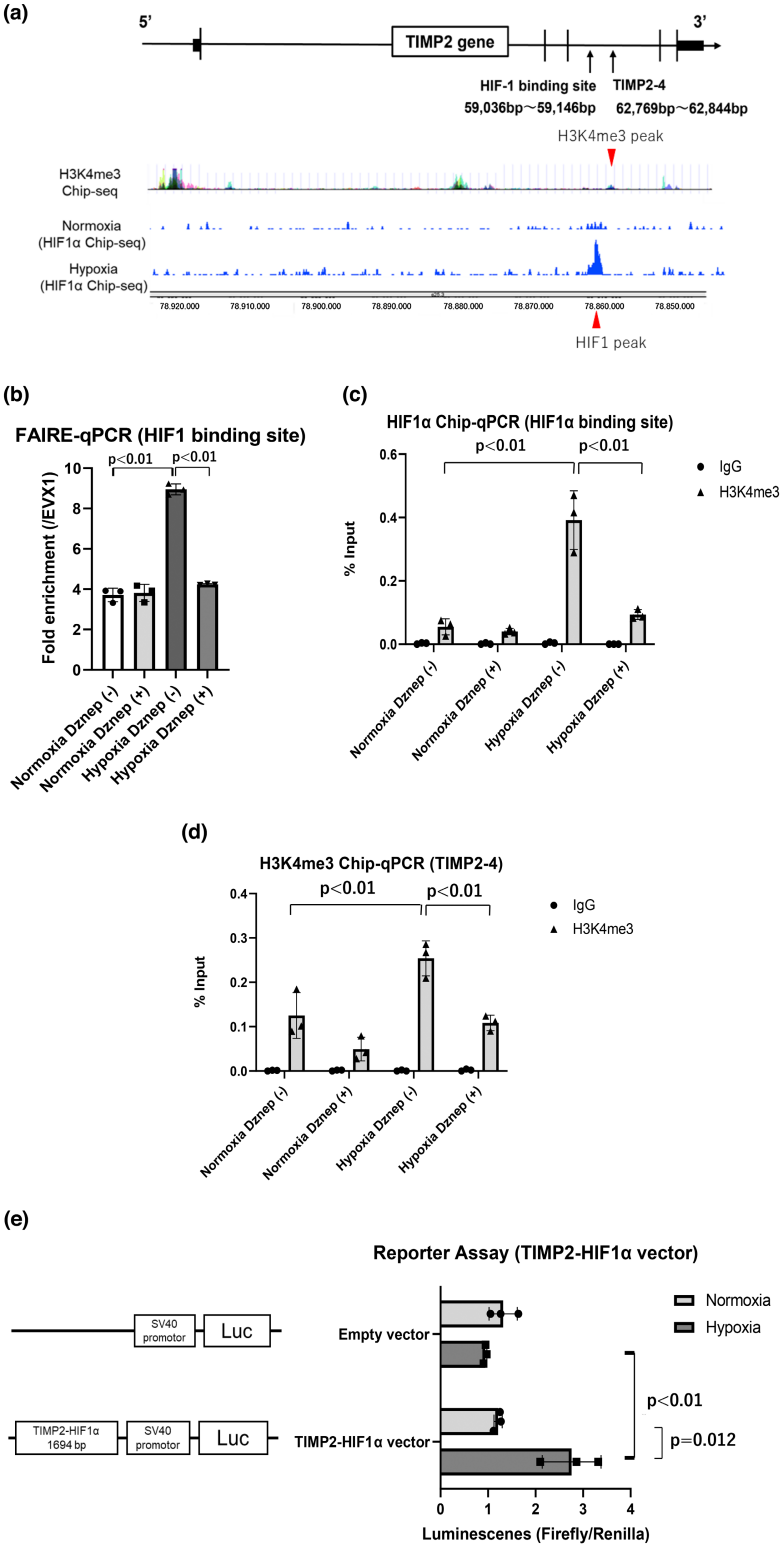
Results of FAIRE‐qPCR, ChIP‐qPCR of HIF1α, and ChIP‐qPCR of H3K4me3 in the HIF1α binding region in the TIMP2 gene in HK‐2 cells under normoxic condition and hypoxic condition for 24 h with or without Dznep treatment (a–d). The target regions of the TIMP2 gene were based on the HIF1α ChIP‐seq data (Kushida et al., 2016) and H3K4me3 ChIP‐seq data from ENCODE (a). These data were based on our group's CHIP‐seq of HIF1α in HK‐2 cells under normoxic and hypoxic conditions and H3K4me3 in seven cell lines under normoxic conditions from ENCODE. The result of FAIRE‐qPCR in the HIF1α binding region (red peak below) was evaluated via fold enrichment in the EVX1 region, which is the negative control region. First bar and plots indicate normoxia without Dznep (3.713 ± 0.336). Second bar and plots indicate normoxia with Dznep (3.811 ± 0.425). Third bar and plots indicate hypoxia without Dznep (8.950 ± 0.273). Fourth bar and plots indicate hypoxia with Dznep (4.235 ± 0.036) (b). The result of ChIP‐qPCR of HIF1α was assessed using %input. First bar and plots indicate normoxia using normal IgG without Dznep (0.00260 ± 0.00229). Second bar and plots indicate normoxia using anti‐H3K4me3 antibody without Dznep (0.0547 ± 0.0252). Third bar and plots indicate normoxia using normal IgG with Dznep (0.00134 ± 0.00232). Fourth bar and plots indicate normoxia using anti‐H3K4me3 antibody with Dznep (0.0404 ± 0.0100). Fifth bar and plots indicate hypoxia using normal IgG without Dznep (0.00353 ± 0.000345). Sixth bar and plots indicate hypoxia using anti‐H3K4me3 antibody without Dznep (0.3919 ± 0.0925). Seventh bar and plots indicate hypoxia using normal IgG with Dznep (0.0000459 ± 0.0000079). Eighth bar and plots indicate hypoxia using anti‐H3K4me3 antibody with Dznep (0.0936 ± 0.0155) (c). The result of ChIP‐qPCR of H3K4me3 in the region (red peak above) near the HIF1α binding site was investigated using %input. First bar and plots indicate normoxia using normal IgG without Dznep (0.00114 ± 0.00099). Second bar and plots indicate normoxia using anti‐H3K4me3 antibody without Dznep (0.125 ± 0.051). Third bar and plots indicate normoxia using normal IgG with Dznep (0.00152 ± 0.00132). Fourth bar and plots indicate normoxia using anti‐H3K4me3 antibody with Dznep (0.049 ± 0.026). Fifth bar and plots indicate hypoxia using normal IgG without Dznep (0.00082 ± 0.00142). Sixth bar and plots indicate hypoxia using anti‐H3K4me3 antibody without Dznep (0.254 ± 0.039). Seventh bar and plots indicate hypoxia using normal IgG with Dznep (0.00226 ± 0.00223). Eighth bar and plots indicate hypoxia using anti‐H3K4me3 antibody with Dznep (0.108 ± 0.017) (d). The results of reporter assay of the pGL3‐promotor vector with the HIF1α binding sequence in TIMP2 (TIMP2‐HIF1α vector). First bar and plots indicate normoxia with empty vector (1.318 ± 0.297). Second bar and plots indicate hypoxia with empty vector (0.940 ± 0.034). Third bar and plots indicate normoxia with TIMP2‐HIF1α vector (1.212 ± 0.086). Fourth bar and plots indicate hypoxia with TIMP2‐HIF1α vector (2.759 ± 0.617) (e). EVX1, even‐skipped homeobox 1; FAIRE, formaldehyde‐assisted isolation of regulatory elements; HIF, hypoxia‐inducible factor; HK‐2, human kidney 2; Luc, luciferase gene; TIMP2, tissue inhibitor of metalloproteinase 2; TSS, transcriptional start site. *p < 0.05, **p < 0.01. Three independent experiments (*n* = 3) were conducted.

Hypoxia‐inducible factor (HIF) is a key transcription factor in response to hypoxia. Whether HIF1α binds to the TIMP2 gene was evaluated. We confirmed that HIF1α bound to the exon region of TIMP2 gene based on our previous HIF1α ChIP‐seq data (Figure 4a) (Kushida et al., 2016). These details were based on our group's CHIP‐seq of HIF1α in HK‐2 cells under normoxic and hypoxic conditions. Results showed that Dznep suppressed the percentage of open chromatin area, which was elevated by hypoxia, via FAIRE‐qPCR in this region (Figure 4b). ChIP‐qPCR of HIF1α showed that HIF1α binding was upregulated by hypoxia and inhibited by Dznep (Figure 4c). The H3K4me3 region (peak 4) near the HIF1α binding region was extracted from the ENCODE data, and we made a primer of TIMP2‐4. ChIP‐qPCR of H3K4me3 was performed in this region (peak 4; TIMP2‐4). ChIP‐qPCR of H3K4me3 was also elevated under hypoxic condition (2.23 ± 0.085 fold) and significantly suppressed (0.38 ± 0.05 fold) by Dznep compared to that without Dznep under hypoxia (Figure 4d).

The gene region with high binding of HIF1α based on the ChIP‐seq data (length: 1694 bp) was cloned into the pGl3‐promotor vector, which contains an SV40 promoter upstream of the luciferase gene, for a dual luciferase reporter assay. Significantly increased luminescence (2.27 ± 0.51 fold) was observed in HK‐2 cells transfected with TIMP2‐HIF1α‐binding DNA inserted into the pGL3‐promotor vector (TH vector) under hypoxic condition, but not in the empty vector (Figure 4e).

## 4 DISCUSSION

**FIGURE 5:**
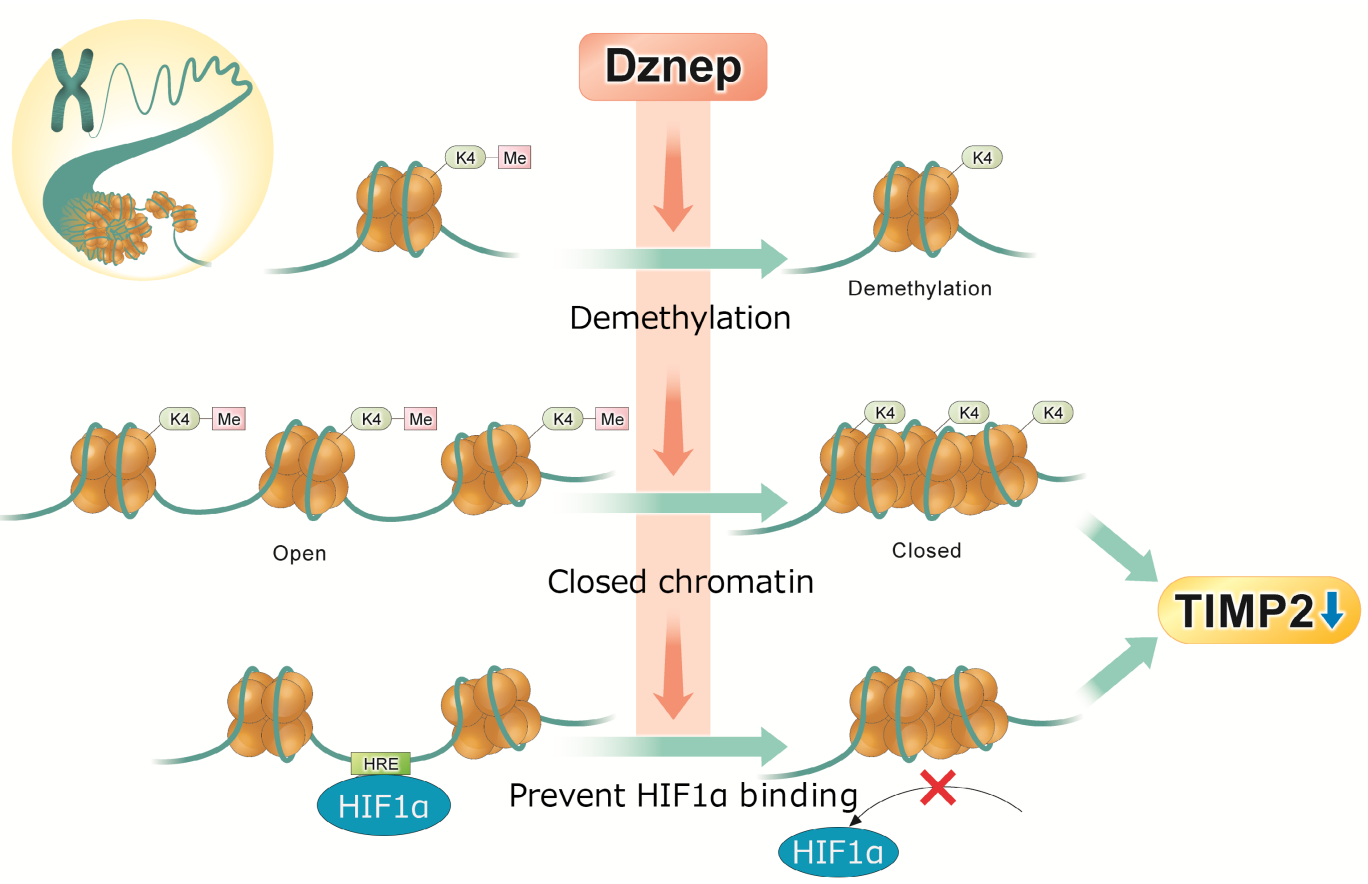
The epigenetic regulation of TIMP2 by Dznep. Dznep promotes demethylation, a closed chromatin structure and prevents HIF1α binding to HREs at the TIMP2 locus. All of these factors prevent the hypoxia associated increase in TIMP2 expression. HIF, hypoxia‐inducible factor; Me, methylation; TIMP2, tissue inhibitor of metalloproteinase 2.

Figure 5 shows a brief summary of this study. The current study showed that Dznep suppressed not only H3K27me3 but also H3K4me3 in HK‐2 cells. In addition, H3K4me3, a histone marker of accelerated expression, was found to be upregulated by hypoxia and downregulated by Dznep in the different gene regions of TIMP2. Similar to H3K4me3, the percentage of open chromatin area was upregulated by hypoxia and downregulated by Dznep. Thus, Dznep suppressed the binding of HIF1α to the TIMP2 gene by reducing open chromatin area.

Dznep decreases the amount of H3K27me3 by suppressing the expression of Ezh2 (Miranda et al., 2009). However, in the current study, inhibiting the expression of Ezh2 did not alter the expression of TIMP2 as did Dznep administration (Glazer et al., 1986; Wang et al., 2020). As shown in Section 1, Dznep inhibits not only Ezh2 but also S‐adenosylhomocysteine hydrolase. Since the inhibition of S‐adenosylhomocysteine hydrolase reduces the methyl groups by suppressing the methionine circuit, we examined the methylation of histones other than H3K27me3 and found that Dznep reduced H3K4me3 in HK‐2 cells. In breast cancer cell lines, Dznep suppressed the methylation of histones other than H3K27me3 (Miranda et al., 2009). In human lung cancer cells, the expression of SET domain containing 1B, a methyltransferase of H3K4, was also regulated by Dznep treatment (Lee & Kim, 2013). Based on a previous study, MM‐102 and OICR‐9429, which are inhibitors of KMT2A, a methyltransferase of H3K4me3, inhibited renal fibrosis in mice with I/R injury (Shimoda et al., 2019).

Dznep suppressed the expression of HIF1α in gastric cancer cells (Huang et al., 2019). Further, the hypoxic induction of two carboxypeptidases (CP) (the CPA4 and CPE gene) was accompanied by the recruitment of HIF1α and upregulation of H3K4me3 at the promoter regions (Moon et al., 2020). The inhibition of histone methylation modification suppressed HIF1α nuclear transport in hepatic stellate cells (Hong et al., 2018). Similar to the current study, a previous report showed that hypoxic stimulation increases the total amount of H3K4me3 in the promoter region of both hypoxia‐activated genes, including early growth response protein 1 and vascular endothelial growth factor, and hypoxia‐repressed genes, such as α‐fetoprotein and albumin (Johnson et al., 2008). Previous studies have shown that the modification of histones (such as H3K4me3) correlated with open chromatin area is induced by hypoxic stimulation and can trigger the HIF1α cascade. Hence, there is a strong association between hypoxia and open chromatin area in the hypoxia‐responsive genes. The result of the current study is consistent with that of previous reports, which showed that hypoxia stimulation in HK‐2 cells increased the amount of H3K4me3, open chromatin area in the TIMP2 gene, and HIF1α binding.

HIF activation has nephroprotective effects in different renal injury models, including I/R injury (Kapitsinou et al., 2012; Koshiji & Huang, 2004; Tanaka, Kojima, et al., 2005; Tanaka, Matsumoto, et al., 2005). A previous study showed worsened renal fibrosis in a group that received long‐term (2–12 weeks) treatment with HIF activators in a 5/6 nephrectomy model (Yu et al., 2012). Furthermore, HIF inhibitors suppressed renal fibrosis in a UUO model (Kimura et al., 2008). Thus, prolonged activation of HIF may have a negative effect on renal fibrosis. Nevertheless, these studies did not examine the expression of TIMP2. However, the current study showed that hypoxia caused increased HIF1α binding in the TIMP2 gene region. Moreover, HIF activation might upregulate the expression of TIMP2 and might be involved in the development of medium‐ to long‐term renal fibrosis. HIF‐prolyl hydroxylase (HIF‐PH) inhibitors can promote the expression of erythropoietin, which is a response to hypoxia. Several large‐phase III randomized controlled trials have shown that HIF‐PH inhibitors are safe and can improve renal anemia. In addition, they are now being marketed for the treatment of renal anemia (Chen et al., 2019; Nangaku et al., 2021, 2022). Since HIF‐PH inhibitors have only been available recently, there are no clinical data on the mid‐ to long‐term effects of HIF activation on CKD progression. If HIF activation contributes to CKD progression, which is the basic experimental information, elevated TIMP2 expression may play an essential role. In hepatocellular carcinoma (HCC) cells, the expression of TIMP2 is suppressed if HIF is activated in a hypoxic environment (Kai et al., 2016). The discrepancy in results may be attributed to differences in cell lines between the current and previous HCC studies and the effect of hypoxia stimulation duration.

The current study had several limitations. First, Dznep had not only the originally predicted repression of H3K27me3 by Ezh2 inhibition but also the repression of H3K4me3. Dznep alters the methylation of histones other than H3K27me3 and gene expression via several complex mechanisms. Therefore, there might be other mechanisms of TIMP2 suppression not associated with HIF1 or open chromatin. This research showed an association between Dznep and open chromatin area and the expression of HIF1α. However, it only showed these associations on the TIMP2 gene only. Thus, we cannot validate the genome‐wide association between Dznep and open chromatin area and the expression of HIF1α. Hence, further studies on this correlation must be conducted. Furthermore, our study examined only HK‐2 cells, and further investigation to elucidate clinical relevance in AKI‐to‐CKD is needed on the mechanism of TIMP2 suppression on the kidney organ.

In conclusion, Dznep reduces the methylation of H3K4me3 and open chromatin area in the TIMP2 gene, thereby inhibiting HIF1α binding and suppressing the expression of TIMP2 caused by hypoxia in HK‐2 cells.

## AUTHOR CONTRIBUTIONS

Tomotaka Yamazaki and Imari Mimura designed research and wrote the original paper; Tomotaka Yamazaki and Imari Mimura conducted the experiments; Yu Kurata, Tetsuhiro Tanaka and  Masaomi Nangaku interpreted the results; Masaomi Nangaku and Tetsuhiro Tanaka provided the conceptual advice; All authors reviewed the manuscript.

## FUNDING INFORMATION

This study was partly supported by the Grant‐in‐Aid for JSPS Fellows (PD) 21J10424 (T.Y.) and for Scientific Research (C) 20K08604 (I.M.) by the Japan Society for the Promotion of Science (JSPS) KAKENHI. This study was also supported by Takeda Science Foundation (I.M.) and Naito Foundation (I.M.). This research was supported by the World‐leading Innovative Graduate Study Program for Life Science and Technology (WINGS‐LST) and by the Ministry of Education, Culture, Sports, Science and Technology (T.Y. and I.M.).

## ETHICS STATEMENT

All the procedures in this paper were in accordance with the Helsinki Declaration of 1975, as revised in 2008.

## Supporting information


Figure S1.
Figure S2.
Figure S3.
