# Supplementary material for: Dznep, a histone modification inhibitor, inhibits HIF1α binding to TIMP2 gene and suppresses TIMP2 expression under hypoxia
Source: Physiol Rep. 2023 Sep 14;11(17):e15810. doi: 10.14814/phy2.15810 (PMC10502026; doi:10.14814/phy2.15810)
Supplement: Supplementary file 1 — Figure S1. Figure S2. Figure S3. [file PHY2-11-e15810-s001.pdf]

Supplementary Figure 1.

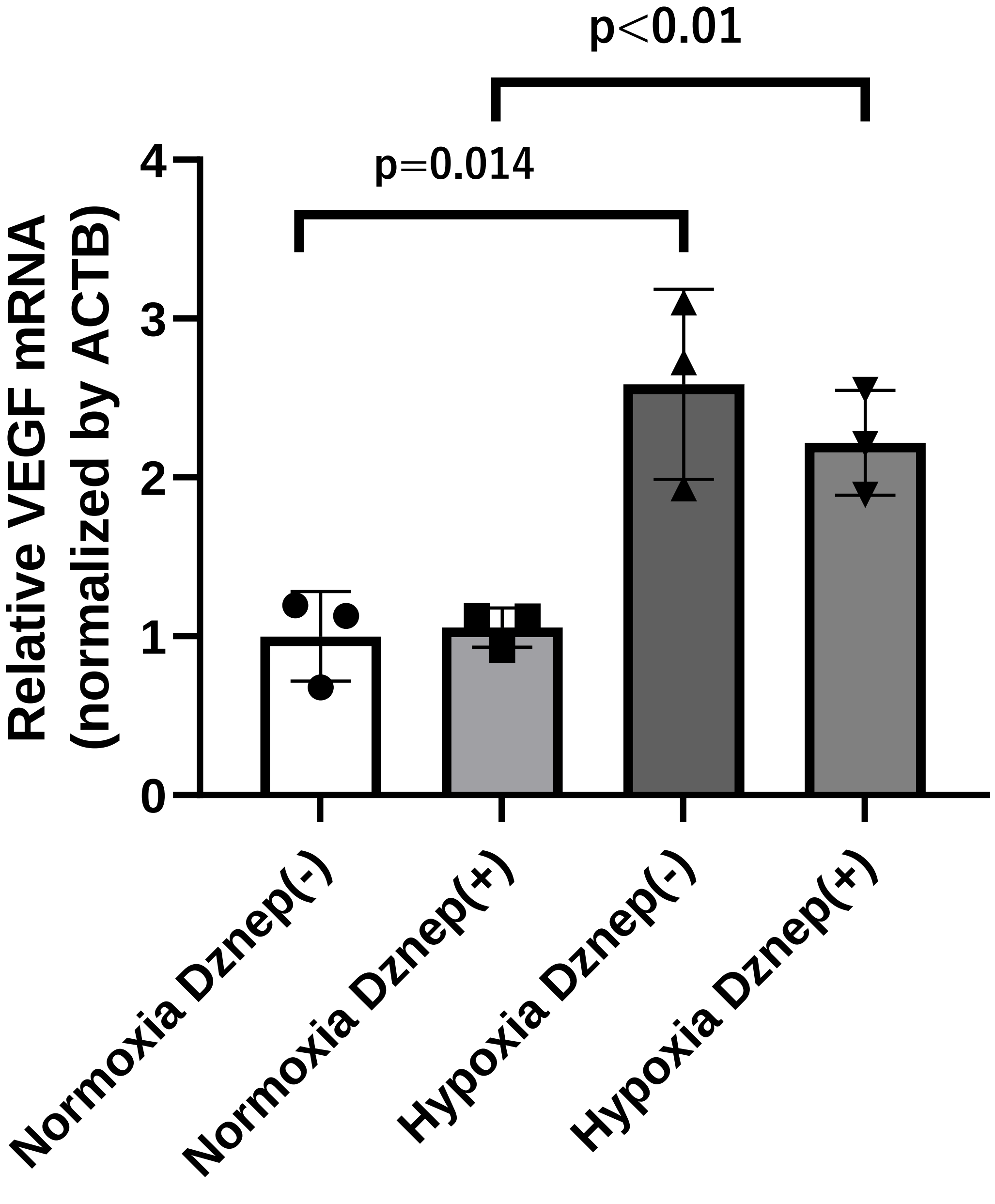

Supplementary Figure 2.

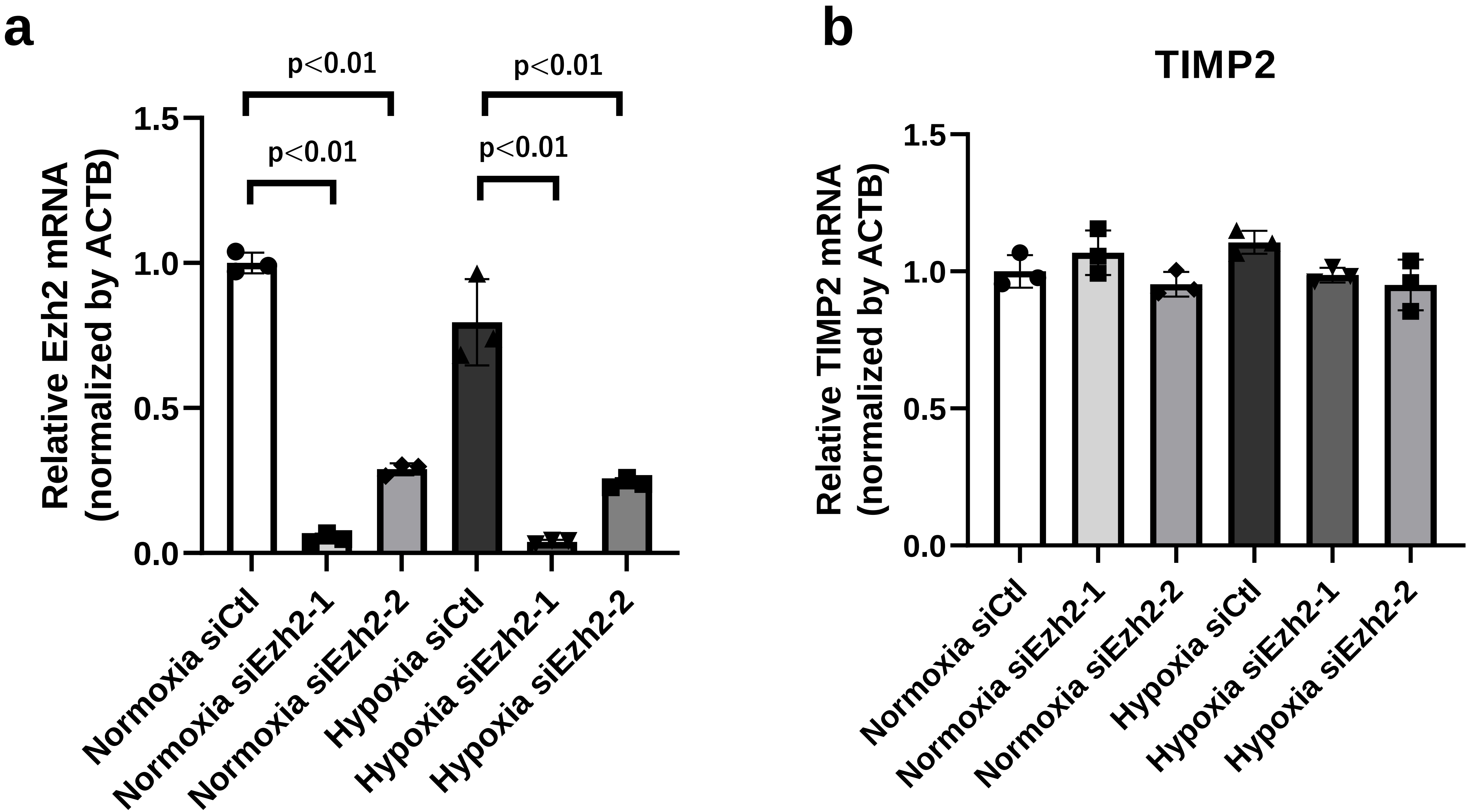

Supplementary Figure 3.

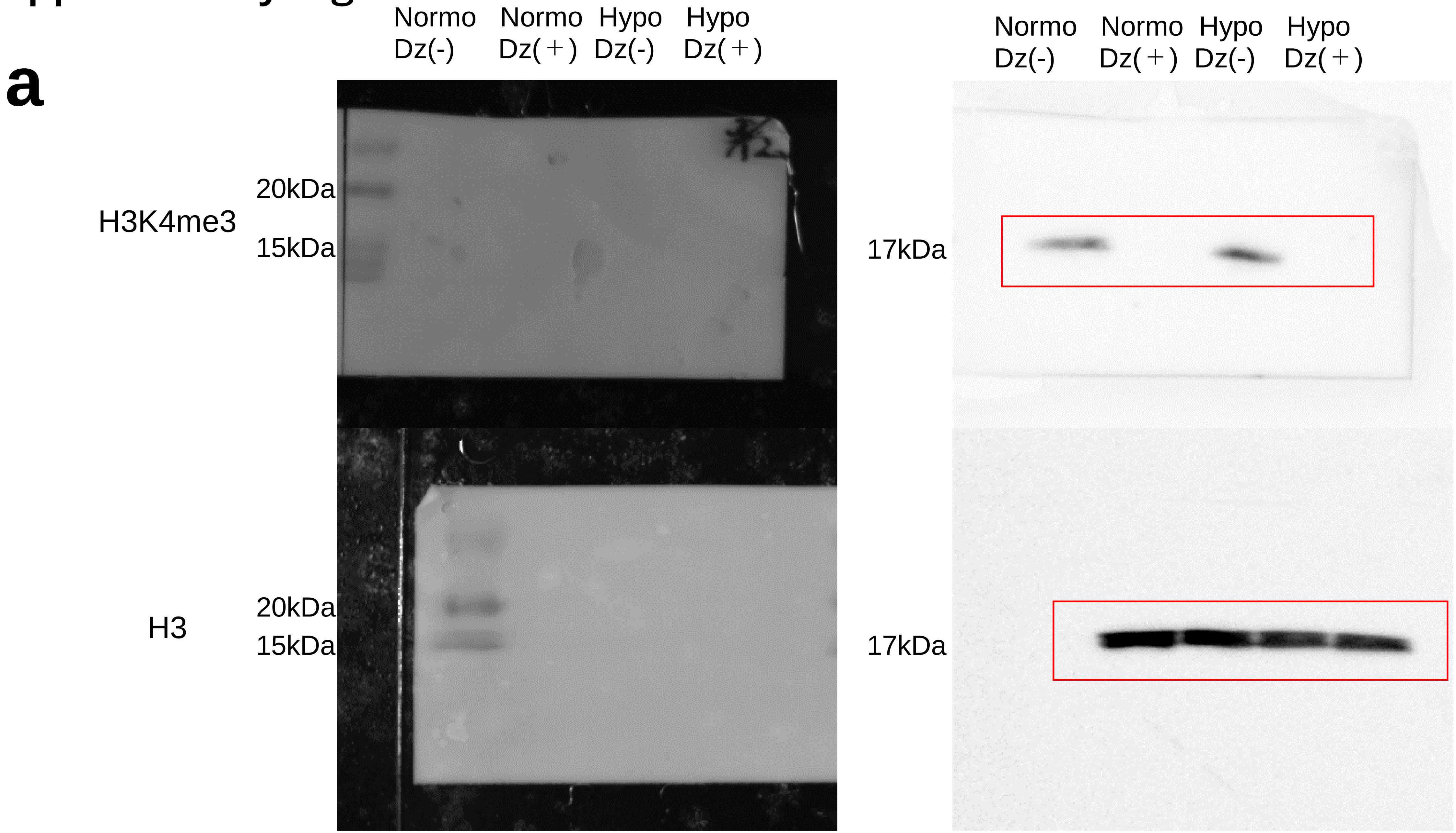

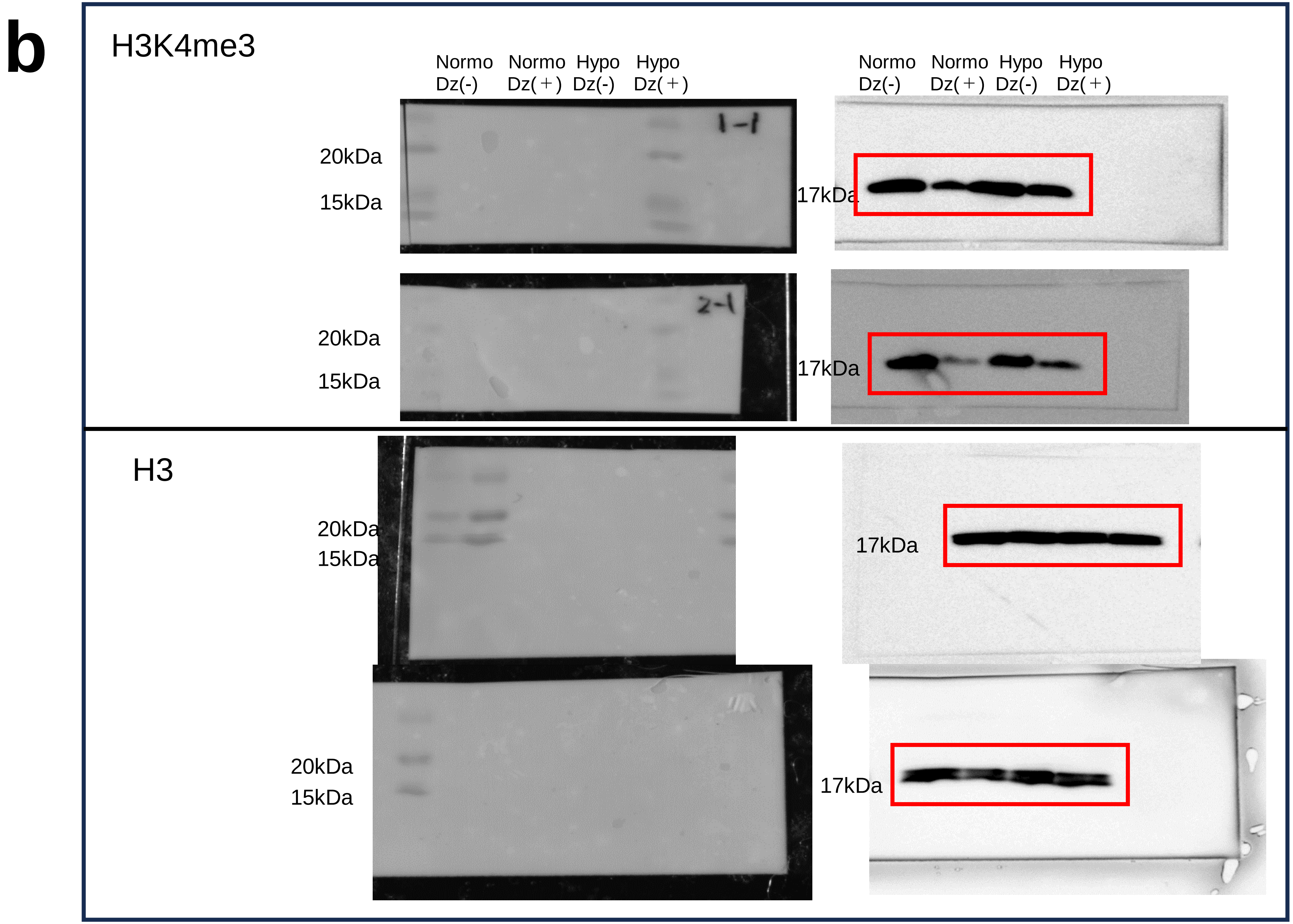

b
H3K4me3
Normo Dz(-)
Normo Dz(+)
Hypo Dz(-)
Hypo Dz(+)
20kDa
15kDa
17kDa
20kDa
15kDa
17kDa
H3
20kDa
15kDa
17kDa
20kDa
15kDa
17kDa
